# Profiles of blood–brain barrier and neurodegeneration markers in cerebrospinal fluid of patients with cerebral amyloid angiopathy

**DOI:** 10.1002/dad2.70221

**Published:** 2025-11-18

**Authors:** João Pinho, Arno Reich, Omid Nikoubashman, Jörg B. Schulz, Kathrin Reetz, Ana Sofia Costa

**Affiliations:** ^1^ Department of Neurology University Hospital RWTH Aachen Aachen Germany; ^2^ Department of Diagnostic and Interventional Neuroradiology University Hospital RWTH Aachen Aachen Germany; ^3^ Department of Diagnostic and Interventional Neuroradiology Evangelisches Klinikum Bethel University Hospital OWL of the University Bielefeld Bielefeld Germany; ^4^ JARA Institute of Molecular Neuroscience and Neuroimaging (INM‐11) Juelich Research Center GmbH and RWTH Aachen University Jülich Germany

**Keywords:** cerebral amyloid angiopathy, cerebrospinal fluid, cognitive impairment, intracerebral hemorrhage

## Abstract

**INTRODUCTION:**

There are few studies analyzing cerebrospinal fluid (CSF) in patients with cerebral amyloid angiopathy (CAA). Our goal was to compare blood–brain barrier and neurodegeneration markers in CSF in CAA patients with and without hemorrhagic markers.

**METHODS:**

In a retrospective study of patients with CAA (Boston criteria version 2.0) identified from the Aachen Memory Database and from in‐hospital admission records, we compared CSF neurodegeneration markers and albumin ratio (a blood–brain barrier permeability marker) in patients with and without hemorrhagic markers.

**RESULTS:**

Among 371 patients with CAA, 113 patients had hemorrhagic markers (30.5%). Lower amyloid beta (Aβ) 42, lower Aβ40, and higher albumin ratio were independently associated with the presence of hemorrhagic markers and an increasing number of lobar microbleeds. Cortical superficial siderosis and a higher imaging burden of CAA were associated with total tau protein.

**DISCUSSION:**

Presence of hemorrhagic markers in CAA patients is associated with lower CSF Aβ42 and Aβ40 and higher blood–brain barrier permeability.

**HIGHLIGHTS:**

- New diagnostic criteria allow for the diagnosis of CAA without hemorrhagic markers.
- CAA hemorrhagic markers are associated with lower Aβ42 and Aβ40 in CSF.
- CAA hemorrhagic markers are associated with higher blood–brain barrier permeability.
- Higher imaging burden of CAA is associated with higher total tau protein in CSF.

## 1 INTRODUCTION

Cerebral amyloid angiopathy (CAA) is a major cause of non‐traumatic lobar intracerebral hemorrhage (ICH), but it is also increasingly recognized as a contributor to cognitive impairment and dementia.^1^, ^2^ Koeman and collaborators recently proposed a pathophysiological framework for the progression of CAA, where initial cerebrovascular deposition of amyloid beta (Aβ) is followed by changes in vascular function, while non‐hemorrhagic and later hemorrhagic cerebral lesions occur in more advanced stages of the disease.^3^ The Boston criteria version 2.0 now allow for the diagnosis of CAA in patients with cognitive presentation and in the absence of hemorrhagic markers.^4^ Previous studies analyzing cerebrospinal fluid (CSF) neurodegeneration markers, namely, Aβ42, Aβ40, total tau, and phosphorylated tau, in CAA patients focused on comparison with patients with Alzheimer´s disease or with healthy controls.^5^ We hypothesized that CSF profiles of neurodegeneration markers and blood–brain barrier permeability in patients with CAA varied according to the presence of hemorrhagic lesions. The goal of this work was to study the profile of CSF neurodegeneration markers and blood–brain barrier function in patients with CAA according to the presence of hemorrhagic markers.

**RESEARCH IN CONTEXT**

1. **Systematic review**: Relevant literature was searched using the terms “cerebral amyloid angiopathy” and “CSF” using PubMed. Previous studies on CSF markers in sporadic CAA patients focused primarily on the comparison with patients with Alzheimer´s disease or with healthy controls. Little is known about blood–brain barrier and neurodegeneration markers in CAA patients according to the presence of hemorrhagic markers.
2. **Interpretation**: This study demonstrates that the presence of hemorrhagic markers (intracerebral hemorrhage, convexity subarachnoid hemorrhage, lobar microbleeds, and/or cortical superficial siderosis) in patients with sporadic CAA (Boston criteria version 2.0) is associated with lower Aβ42 and lower Aβ40 in CSF and higher blood–brain barrier permeability. Higher CAA burden is associated with higher CSF total tau protein. This supports the hypothesis that hemorrhagic lesions occur late in the pathophysiological timeline of CAA.
3. **Future directions**: Longitudinal data to understand clinical trajectories of CAA patients without hemorrhagic markers and predictors of progression are needed.

## 2 METHODS

### 2.1 Study population

We conducted a retrospective, cross‐sectional, single‐center study of patients with sporadic CAA. We reviewed consecutive patients from the Aachen Memory Database (2009 to 2019) with available CSF neurodegeneration markers, brain magnetic resonance imaging (MRI), and comprehensive neuropsychological assessment and included patients who fulfilled the Boston criteria version 2.0 for CAA. All patients had been referred for diagnostic evaluation of suspected cognitive impairment. Details of clinical and neuropsychological assessment and of MRI rating can be found elsewhere.^6^ Patients with inflammatory or infectious diseases of the central nervous system (CNS), intracranial tumor, traumatic brain injury, primary or secondary vasculitis of CNS, genetic causes for cognitive impairment, frontotemporal dementia, primary progressive aphasia, Parkinson´s disease, progressive supranuclear palsy, corticobasal syndrome, Lewy body disease, Huntington´s disease, and normal pressure hydrocephalus were excluded. We specifically did not exclude patients who had concomitant criteria for Alzheimer´s disease. Additionally, we performed a query to identify all hospitalized patients in our Department of Neurology between 2018 and 2024, who had a main or secondary diagnosis of cerebral amyloid angiopathy according to the I68.0 code of the International Classification of Diseases and Related Health Problems, 10th Edition—Clinical Modifications (ICD‐10‐CM). Duplicated patients, patients with CAA‐related inflammation, hereditary CAA, iatrogenic CAA, unavailable neurodegeneration markers in CSF, unavailable MRI, and those who did not fulfill the Boston criteria version 2.0 were excluded. Patients who had concomitant Alzheimer´s disease, defined according to the IWG recommendation,^7^ were identified and not excluded from the study.

Baseline demographic and clinical characteristics, cognitive assessment results (CERAD‐NAB+ and Mini‐Mental State Examination [MMSE]), laboratory blood parameters, and results of CSF parameters were collected from the Aachen Memory Database or from the individual patient records. The CAA small vessel disease (CAA‐SVD) score was calculated as previously described.^8^ CSF samples and blood samples were collected on the same day. All CSF samples were analyzed for neurodegeneration markers at the University Medical Center Göttingen Neurochemical Laboratory using commercially available assays that have been validated in clinical populations. Enzyme‐linked immunosorbent assays were used for the quantitative determination of Aβ42 (INNOTEST^®^ β‐AMYLOID [1‐42], Fujirebio Europe NV), Aβ40 (amyloid‐beta [1‐40] CSF ELISA [RE59651], IBL International GmbH), total tau protein (INNOTEST^®^ hTau Ag, Fujirebio Europe NV), and phosphorylated tau fractions (INNOTEST^®^ PHOSPHO‐17 TAU [181P], Fujirebio Europe NV) in CSF, according to each manufacturer's instructions. The albumin ratio was calculated using the following formula: (CSF albumin/serum albumin) × 1000. The upper normal limit of albumin ratio for each patient was calculated using the following formula: [(age at lumbar puncture/15) + 4].^9^ We calculated composite scores for a priori defined cognitive domains by averaging individual *z*‐scores on the CERAD‐NAB+ based on published normative data,^10^ adjusted for age, education, and sex. Cognitive domains included attention (Trail Making Test A [TMT Part A]), executive function (TMT Part B, phonemic fluency), language (naming, semantic fluency), memory (verbal learning, verbal recall, verbal savings and verbal recognition, non‐verbal recall), and visuoconstruction (figure copy). We calculated the frequency of patients impaired in each cognitive domain, using a *z*‐score of −1.5 as a cut‐off.

### 2.2 Statistical analysis

We grouped our final study population according to the presence of hemorrhagic markers before or at the time of lumbar puncture (hemorrhage− or hemorrhage+). Hemorrhagic markers were defined as ICH, acute convexity subarachnoid hemorrhage, lobar microbleed, and/or cortical superficial siderosis (cSS). We further divided the hemorrhage+ group into two groups: those with possible CAA and those with probable CAA, according to the modified Boston criteria version 1.5.^11^ Baseline demographic, clinical, imaging, and CSF characteristics were compared using chi‐squared tests, Fisher exact tests, and Mann–Whitney tests as adequate. We performed Spearman correlations between age and each of the CSF parameters. To adjust for the influence of age on possible associations of CSF parameters with the presence of hemorrhagic markers, we performed multivariable logistic regressions with the presence of hemorrhagic markers as the dependent variable and each CSF parameter and age as independent variables. We carried out the same regression analyses specific for the presence of lobar microbleeds, for the presence of cSS, and for the categorized CAA‐SVD score (≥3) as the dependent variables. To study the relationship between the severity of lobar microbleeds and each of the CSF parameters, we performed linear regression analyses with the number of lobar microbleeds as the dependent variable and included age as a covariate. Adjusted odds ratio (aOR), adjusted *ß*‐coefficients, and respective 95% confidence intervals (95%CI) were calculated. aOR and adjusted *ß*‐coefficient for albumin ratio were additionally adjusted for serum albumin. We conducted two sensitivity analyses. To exclude a possible bias of concomitant Alzheimer´s disease, we excluded these patients from the multivariable logistic regression analyses and from the linear regression analyses. To exclude a possible bias related to the presence of antithrombotic therapy, we additionally adjusted the multivariable logistic analyses by including antithrombotic therapy as an independent variable. The threshold for statistical significance was set at an alpha value of 0.05. The study was approved by the local ethics committee (EK 018‐19) and carried out in accordance with the Code of Ethics of the World Medical Association (Declaration of Helsinki). The study was conducted according to the Strengthening the Reporting of Observational Studies in Epidemiology (STROBE) recommendations.^12^ No method for imputing missing data was used; for each analysis, only patients without missing data were included. Data will be made available on reasonable request for research purposes and according to the local guidelines.

## 3 RESULTS

**FIGURE 1:**
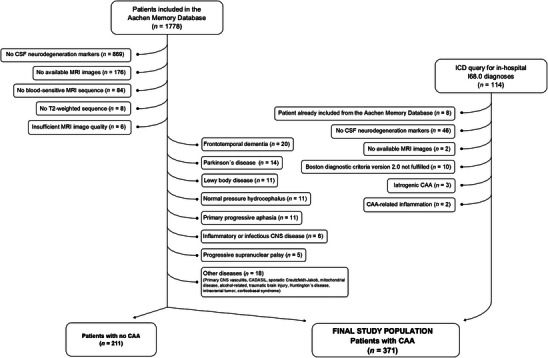
Patient flow chart.

Among 1778 consecutive patients included in the Aachen Memory Database, we selected 328 patients with sporadic CAA. Among 114 patients identified by the diagnosis query, we additionally selected 43 patients with sporadic CAA (Figure 1). The final study population consisted of 371 patients with sporadic CAA (possible CAA = 265, probable CAA = 106), with a median age of symptom onset of 71 years (interquartile range [IQR] 65 to 76) and median age at lumbar puncture of 73 years (IQR 67 to 78). One hundred ninety patients were female (51.2%). Most of the patients had cognitive impairment as the first clinical manifestation (*n* = 342, 92%), followed by symptomatic ICH or acute convexity subarachnoid hemorrhage (*n* = 23, 6.2%) and transient focal neurological episodes without acute intracranial hemorrhage (*n* = 4, 1.1%).

### 3.1 Comparison of CAA patients with and without hemorrhagic markers

**FIGURE 2:**
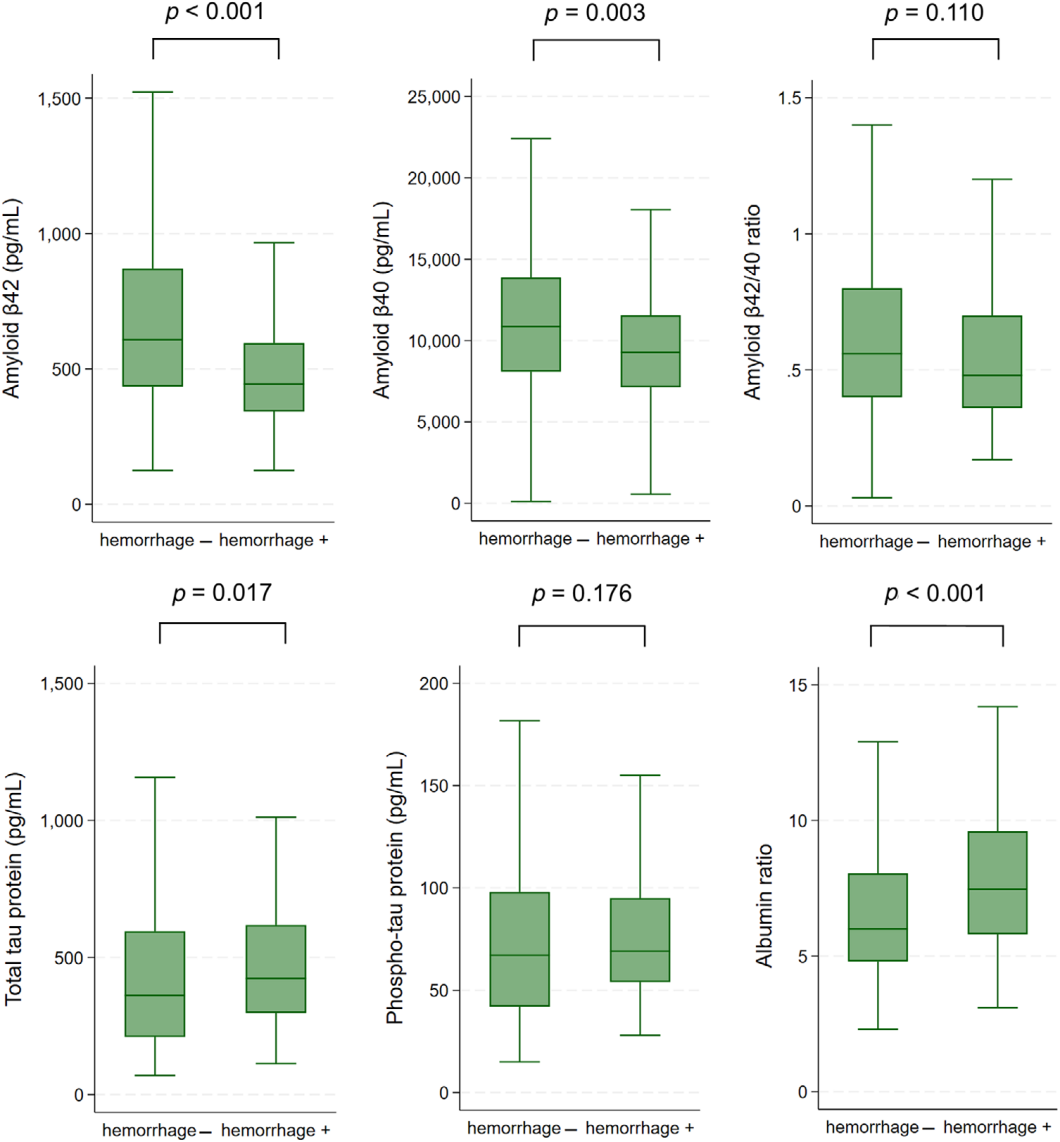
Distribution of cerebrospinal fluid (CSF) parameters based on presence of hemorrhagic markers.

Characteristics of the study population according to the presence of hemorrhagic markers are presented in Table 1. Patients with hemorrhagic markers were older at first manifestation (75 vs 69 years, *p* < 0.001), older at lumbar puncture (76 vs 71 years, *p* < 0.001), had more frequently a history of previous ischemic stroke (20.4% vs 8.1%, *p* < 0.001), had more frequently a multispot pattern of white matter hyperintensities (63.7% vs 52.7%, *p* = 0.049), and had higher CAA‐SVD scores (median 1 vs 2, *p* < 0.001). Patients with hemorrhagic markers had lower platelet counts (227 vs 249 /nL, *p* = 0.018) and lower serum albumin (4000 vs 4200 mg/dL, *p* = 0.003). Lumbar puncture was performed later after symptom onset in patients with hemorrhagic markers (19 vs 25 months, *p* = 0.023). However, among the 27 patients who had ICH/acute convexity subarachnoid hemorrhage before lumbar puncture, the majority of patients underwent lumbar puncture in the acute phase after hemorrhage (IQR 0 to 4 months). Patients with hemorrhagic markers had lower Aβ42 levels (444 vs 608 pg/mL, *p* < 0.001), lower Aβ40 levels (9271 vs 10,872 pg/mL, *p* = 0.003), higher total tau protein (424 vs 362 pg/mL, p = 0.017) and higher albumin ratio (7.5 vs 6.0, *p* < 0.001) (Figure 2). Twenty‐one percent of patients without hemorrhagic markers had an albumin ratio above the upper limit for age, in comparison with 28% of those with hemorrhagic markers (*p* = 0.192). Some of these statistically significant associations did not persist when we compared CAA patients without hemorrhagic markers (hemorrhage−) with patients with possible CAA (Boston criteria version 1.5), namely: presence of a multispot pattern of white matter hyperintensities and all of the CSF parameters (Aβ42, Aβ40, total tau protein, albumin ratio) (Table 1). However, these associations remained significant when comparing patients without hemorrhagic markers (hemorrhage−) with patients with probable CAA (Boston criteria version 1.5) (Table 1). The prevalence of Alzheimer's disease was higher in the group of possible CAA (38.8%) and lower in the group of probable CAA (12.5%), in comparison with patients with CAA with no hemorrhagic markers (24.4%, *p* = 0.037 and 0.040, respectively). Patients with probable CAA less frequently were treated with antithrombotic medications at the time of diagnosis in comparison to CAA patients without hemorrhagic markers (28.1% vs 44.6%, *p* = 0.017), and platelet count was similar between these two groups of patients (249 vs 240 /nL, p = 0.329). Except for the Aβ40 and albumin ratio, all other analyzed CSF parameters were correlated with age (Table S1).

**TABLE 1:** Baseline characteristics of study population based on presence of hemorrhagic markers and diagnosis according to modified Boston criteria (version 1.5) for cerebral amyloid angiopathy.

	CAA hemorrhage− (*n* = 258)	CAA hemorrhage+ (*n* = 113)	*P*	Possible CAA version 1.5 (*n* = 49)	*P*	Probable CAA version 1.5 (*n* = 64)	*P*
Age at first manifestation (years)	69 (62 to 75)	75 (70 to 78)	<0.001	76 (70 to 78)	<0.001	74 (70 to 78)	<0.001
Age at lumbar puncture (years)	71 (65 to 77)	76 (72 to 79)	<0.001	76 (72 to 80)	<0.001	76 (72 to 79)	<0.001
Female sex	141 (54.7)	49 (43.4)	0.045	20 (40.8)	0.075	29 (45.3)	0.180
**Vascular risk factors and comorbidities**							
Arterial hypertension	166 (64.3)	74 (65.5)	0.832	27 (55.1)	0.220	47 (73.4)	0.169
Diabetes	45 (17.4)	17 (15.0)	0.569	6 (12.2)	0.370	11 (17.2)	0.962
Dyslipidemia	114 (44.2)	59 (52.2)	0.154	27 (55.1)	0.160	32 (50.0)	0.403
Current or past smoking	70 (27.1)	21 (18.6)	0.078	11 (22.4)	0.495	10 (15.6)	0.057
Atrial fibrillation	12 (4.7)	10. (8.0)	0.115	4 (8.2)	0.311	6 (9.4)	0.141
Coronary heart disease	17 (6.6)	11 (9.7)	0.291	6 (12.2)	0.168	5 (7.8)	0.782
Previous ischemic stroke	21 (8.1)	23 (20.4)	<0.001	11 (22.4)	0.003	12 (18.8)	0.012
Alzheimer´s disease	63 (24.4)	27 (23.9)	0.914	19 (38.8)	0.037	8 (12.5)	0.040
**Antithrombotic medication** ^a^	115 (44.6)	41 (36.3)	0.137	23 (46.9)	0.760	18 (28.1)	0.017
**First manifestation**			<0.001		0.002		<0.001
Cognitive impairment	258 (100)	84 (75.7)		48 (98.0)		36 (58.1)	
Transient focal neurological episodes	0	4 (3.6)		1 (2.0)		3 (4.8)	
ICH/cSAH	0	23 (20.7)		0		23 (37.1)	
**MRI findings**							
Severe burden of EPVS in CSO	200 (77.5)	82 (72.6)	0.304	33 (67.3)	0.127	49 (76.6)	0.870
Multispot WMH pattern	136 (52.7)	72 (63.7)	0.049	28 (57.1)	0.569	44 (68.8)	0.021
Lobar microbleeds	0	100 (88.5)	<0.001	40 (81.6)	<0.001	60 (93.8)	<0.001
Number of lobar microbleeds	–	1 (1 to 6)	–	1 (1 to 1)	–	5 (2 to 21)	–
Cortical superficial siderosis	0	42 (37.2)	<0.001	8 (16.3)	<0.001	34 (53.1)	<0.001
CAA‐SVD score	1 (1‐1)	2 (2 to 4)	<0.001	1 (1 to 2)	<0.001	4 (3 to 5)	<0.001
**Laboratory blood parameters**							
Platelet count (/nL)	249 (203 to 289)	227 (192 to 279)	0.018	226 (184 to 258)	0.005	240 (193 to 294)	0.329
Alanine aminotransferase (U/L)	20 (16 to 25)	19 (15 to 25)	0.472	19 (15 to 26)	0.769	19 (15 to 25)	0.441
Aspartate aminotransferase (U/L)	24 (20 to 28)	24 (19 to 29)	0.458	24 (20 to 29)	0.553	24 (19 to 28)	0.580
Gamma‐glutamyl transferase (U/L)	22 (16 to 32)	22 (16 to 29)	0.480	22 (16 to 27)	0.469	23 (15 to 30)	0.697
Glomerular filtration rate (ml/min/1.73m^2^)	62 (60 to 82)	73 (59 to 85)	0.356	67 (58 to 83)	0.551	74 (60 to 86)	0.064
Serum albumin (mg/dL)	4200 (3930 to 4400)	4000 (3760 to 4230)	0.003	4000 (3760 to 4200)	0.023	3995 (3745 to 4253)	0.019
**CSF parameters**							
Time between first symptoms and lumbar puncture (months)	19 (12 to 27)	25 (12 to 42)	0.023	25 (12 to 47)	0.019	24 (12 to 40)	0.253
Amyloid β42 (pg/mL)	608 (435 to 873)	444 (343 to 605)	<0.001	525 (387 to 806)	0.117	424 (331 to 534)	<0.001
Amyloid β40 (pg/mL) ^b^	10872 (8101 to 13881)	9271 (7096 to 11581)	0.003	10889 (8048 to 13220	0.860	8879 (6843 to 10534)	<0.001
Amyloid β42/40 ratio^b^	0.56 (0.40 to 0.80)	0.48 (0.36 to 0.70)	0.110	0.48 (0.35 to 0.81)	0.227	0.49 (0.37 to 0.69)	0.206
Total tau protein (pg/mL)	362 (208 to 598)	424 (297 to 620)	0.017	382 (259 to 611)	0.258	475 (318 to 637)	0.014
Phosphorylated tau (pg/mL)	67 (42 to 98)	69 (53 to 96)	0.176	69 (48 to 96)	0.640	73 (56 to 96)	0.126
Albumin ratio^c^	6.0 (4.8 to 8.1)	7.5 (5.8 to 9.6)	<0.001	6.9 (5.5 to 8.5)	0.051	7.6 (5.9 to 10.0)	<0.001
Albumin ratio above upper limit for age^c^	42 (20.6)	24 (27.6)	0.192	8 (22.9)	0.760	16 (30.8)	0.117

*Note*: Data are presented as n (%) or median (interquartile range). All comparisons were performed using the group “CAA hemorrhage‐” as reference.

Abbreviations: CAA, cerebral amyloid angiopathy; CSO, centrum semiovale; CSF, cerebrospinal fluid; EPVS, enlarged perivascular spaces; ICH/cSAH, intracerebral hemorrhage and/or convexity subarachnoid hemorrhage; MRI, magnetic resonance imaging; WMH, white matter hyperintensities.

^a^ Antiplatelets and/or anticoagulants at the time of diagnosis.

^b^ Available for 333 patients.

^c^ Available for 291 patients.

The median cognitive composite scores show a largely inconspicuous to mildly impaired performance in all groups. The group of patients with hemorrhagic markers less frequently had impairment of episodic memory (33.7% vs 47.0%, *p* = 0.026), and the exclusion of patients with Alzheimer's disease did not change this association. There were no significant differences in cognitive performance between the group without hemorrhagic markers and the group with possible CAA (Boston criteria version 1.5). Patients with probable CAA (Boston criteria version 1.5) had higher episodic memory composite scores (median −1.5 vs −1.8, *p* = 0.047) and less frequently presented episodic memory impairment (30.5% vs 47.0%, *p* = 0.023) in comparison to patients without hemorrhagic markers (Table 2).

**TABLE 2:** Cognitive performance according to the presence of hemorrhagic markers.

	CAA hemorrhage−	n	CAA hemorrhage+	*n*	p	Possible CAA v1.5	*n*	*p*	Probable CAA v1.5	*n*	*p*
**Global cognitive status**											
MMSE	26 (21 to 28)	242	25 (22 to 27)	107	0.697	25 (21 to 28)	44	0.958	26 (23 to 27)	63	0.618
**Composite scores**											
Attention	−1.4 (‐2.5 to ‐0.6)	222	−1.3 (‐2.0 to ‐0.7)	99	0.644	−1.4 (‐2.5 to ‐0.8)	40	0.852	−1.3 (‐2.0 to ‐0.6)	59	0.431
Executive functions	−0.9 (‐1.7 to ‐0.2)	213	−1.0 (‐1.7 to ‐0.4)	94	0.569	−1.1 (‐1.9 to ‐0.2)	39	0.421	−0.9 (‐1.4 to ‐0.4)	55	0.888
Language	−0.9 (‐1.8 to ‐0.1)	224	−0.8 (‐1.7 to ‐0.3)	101	0.890	−0.8 (‐1.9 to ‐0.4)	41	0.793	−0.8 (‐1.6 to 0)	60	0.688
Visuoconstruction	−0.9 (‐2.3 to 0.6)	221	−0.6 (‐1.9 to 0.9)	98	0.135	−0.1 (‐1.9 to 0.9)	39	0.159	−0.8 (‐2.0 to 1.0)	59	0.341
Episodic memory	−1.8 (‐2.8 to ‐0.7)	219	−1.6 (‐2.4 to ‐0.8)	98	0.193	−1.9 (‐2.7 to ‐0.9)	39	0.806	−1.5 (‐2.2 to ‐0.6)	59	0.047
**Presence of impairment**											
Attention	80 (36.0)	222	28 (28.3)	99	0.175	13 (32.5)	40	0.667	15 (25.4)	59	0.126
Executive functions	49 (23.0)	213	18 (19.1)	94	0.451	9 (23.1)	39	0.992	9 (16.4)	55	0.286
Language	51 (22.8)	224	23 (22.8)	101	0.999	10 (24.4)	41	0.821	13 (21.7)	60	0.856
Visuoconstruction	66 (29.9)	221	23 (23.5)	98	0.240	8 (20.5)	39	0.233	15 (25.4)	59	0.504
Episodic memory	103 (47.0)	219	33 (33.7)	98	0.026	15 (38.6)	39	0.322	18 (30.5)	59	0.023

*Note*: Data presented as *n* (%) or median (interquartile range). All comparisons were performed using the group “CAA hemorrhage−” as reference.

Abbreviation: CAA, cerebral amyloid angiopathy; MMSE, Mini‐Mental State Examination.

### 3.2 Association of CSF markers and hemorrhagic markers

After adjustment, Aβ42 (aOR per 100‐pg/mL decrease 1.13, 95% CI 1.04 to 1.24, *p* = 0.004), Aβ40 (aOR per 1000‐pg/mL decrease 1.09, 95% CI 1.03 to 1.15, *p* = 0.002), and albumin ratio (aOR per 1‐point increase 1.11, 95% CI 1.02 to 1.20, *p* = 0.014) remained independently associated with the presence of hemorrhagic markers (Table 3). The multivariable regression analyses for the association of CSF markers with the presence of lobar microbleeds did not show any significant association (Table 3). After adjustments, significant associations were found between cSS and Aβ42 (aOR per 100‐pg/mL decrease 1.08, 95% CI 1.03 to 1.14, *p* = 0.003), Aβ40 (aOR per 1000‐pg/mL decrease 1.34, 95% CI 1.19 to 1.48, *p* < 0.001), Aβ42/40 ratio (aOR per 0.1‐point decrease 0.92, 95% CI 0.86 to 0.99, *p* = 0.022), total tau protein (aOR per 100‐pg/mL increase 1.12, 95% CI 1.03 to 1.23, *p* = 0.009), and albumin ratio (aOR per 1‐point increase 1.15, 95% CI 1.05 to 1.26, *p* = 0.004) (Table 3). Additionally, we found an independent association of a higher CAA‐SVD score (≥3) with Aβ42 (aOR per 100‐pg/mL decrease 1.40, 95% CI 1.20 to 1.63, *p* < 0.001), Aβ40 (aOR per 1000‐pg/mL decrease 1.24, 95% CI 1.13 to 1.35, *p* < 0.001), total tau protein (aOR per 100‐pg/mL increase 1.12, 95% CI 1.03 to 1.22, *p* = 0.007), and albumin ratio (aOR per 1‐point increase 1.15, 95% CI 1.05 to 1.27, *p* = 0.003) (Table 3).

**TABLE 3:** Multivariable logistic regression analyses with presence of hemorrhagic markers, presence of cerebral lobar microbleeds, presence of cortical superficial siderosis, and CAA‐SVD score ≥ 3 as the dependent variables.

	Hemorrhage+		Lobar microbleeds		Cortical superficial siderosis		CAA‐SVD score ≥ 3	
	aOR (95%CI)	*P*	aOR (95%CI)	*P*	aOR (95%CI)	*P*	aOR (95%CI)	*P*
Aβ42 (per 100‐pg/mL decrease)^a^	1.13 (1.04 to 1.24)	0.004	1.08 (1.00 to 1.18)	0.067	1.08 (1.03 to 1.14)	0.003	1.40 (1.20 to 1.63)	<0.001
Aβ40 (per 1000‐pg/mL decrease)^a^	1.09 (1.03 to 1.15)	0.002	1.04 (0.99 to 1.10)	0.118	1.34 (1.19‐1.48)	<0.001	1.24 (1.13 to 1.35)	<0.001
Aβ42/40 ratio (per 0.1‐point decrease)^a^	0.99 (0.93 to 1.05)	0.754	1.02 (0.95 to 1.09)	0.553	0.92 (0.86 to 0.99)	0.022	0.96 (0.90 to 1.03)	0.239
Total tau protein (per 100‐pg/mL increase)^a^	1.05 (0.98 to 1.12)	0.210	0.99 (0.92‐1.07)	0.805	1.12 (1.03 to 1.23)	0.009	1.12 (1.03 to 1.22)	0.007
Phosphorylated tau (per 10‐pg/mL increase)^a^	1.07 (0.59 to 1.93)	0.824	0.96 (0.52 to 1.76)	0.883	1.58 (0.71 to 3.54)	0.262	1.50 (0.72 to 3.15)	0.280
Albumin ratio (per 1‐point increase)^b^	1.11 (1.02 to 1.20)	0.014	1.07 (0.99 to 1.16)	0.100	1.14 (1.03 to 1.26)	0.011	1.16 (1.05 to 1.27)	0.003

Abbreviations: aOR, adjusted odds ratio; CI, 95% confidence interval.

^a^ Adjusted for age at lumbar puncture.

^b^ Adjusted for age at lumbar puncture and serum albumin.

The exclusion of patients with concomitant Alzheimer's disease resulted in some changes in these results (Table S2): Total tau protein was independently associated with the presence of hemorrhagic markers (aOR per 1000‐pg/mL increase 1.13, 95% CI 1.03 to 1.25, *p* = 0.014); there was a loss of the significant association between Aβ42/40 ratio and cSS; both cSS and a higher CAA‐SVD score (≥3) were associated with higher levels of phosphorylated tau protein (aOR per 10‐pg/mL increase 2.95, 95% CI 1.06 to 8.20, *p* = 0.038 and 3.05, 95% CI 1.18 to 7.85, *p* = 0.021, respectively).

The inclusion of antithrombotic medication in the multivariable logistic regression models as an independent variable did not change the results significantly (Table S3).

### 3.3 Association of CSF markers with the number of lobar microbleeds

Although the presence of lobar microbleeds was not associated with CSF parameters, an increasing number of lobar microbleeds was associated with lower Aβ42 (age‐adjusted *ß*‐coefficient per 100‐pg/mL‐decrease 0.11, 95% CI 0.05 to 2.67, *p* = 0.042), lower Aβ40 (age‐adjusted *ß*‐coefficient per 1000‐pg/mL decrease 0.14, 95% CI 0.30 to 2.21, *p* = 0.010), and higher albumin ratio (adjusted *ß*‐coefficient per 1‐point increase 0.12, 95% CI = 0.05 to 3.29, *p* = 0.044), but not with other CSF parameters. After exclusion of patients with concomitant Alzheimer's disease, the number of lobar microbleeds remained independently associated with Aβ42 and Aβ40 (age‐adjusted *ß*‐coefficient per 100‐pg/mL decrease in Aβ42 0.14, 95% CI 0.25 to 3.49, *p* = 0.024; age‐adjusted *ß*‐coefficient per 1000‐pg/mL decrease in Aβ40 0.14, 95% CI 0.15 to 2.83, *p* = 0.029), but not with albumin ratio (adjusted *ß*‐coefficient per 1‐point increase in albumin ratio 0.12, 95% CI = −0.22 to 4.20, *p* = 0.077).

## 4 DISCUSSION

We demonstrate in this study that among patients with sporadic CAA, patients who have hemorrhagic markers have lower levels of Aβ42 and Aβ40 in CSF in comparison to patients without hemorrhagic markers. Concerning the role of specific hemorrhagic markers, we found that an increasing number of lobar microbleeds, but not their mere presence, was independently associated with lower CSF levels of Aβ42 and Aβ40. At the same time, the presence of cSS was robustly associated with lower CSF levels of Aβ42 and Aβ40. We also demonstrate that a higher imaging burden of CAA is associated with lower CSF levels of Aβ42 and Aβ40. The fact that patients with possible CAA according to the modified Boston criteria version 1.5, who typically have a low burden of hemorrhagic lesions, have similar Aβ42 and Aβ40 levels in CSF in comparison to patients without hemorrhagic markers, supports the validity of the results. These findings are in line with the results of a study with patients with Dutch‐type hereditary CAA, in which patients with symptomatic hemorrhage had lower CSF levels of both Aβ42 and Aβ40 than presymptomatic carriers.^13^ In another study with sporadic CAA patients, the presence of cSS was associated with lower CSF levels of Aβ42 and a lower CSF Aβ42/40 ratio.^14^ Additionally, higher CSF neurofilament light chain levels were previously associated not only with increasing neuroimaging severity of CAA but also with lower Aβ42 and lower Aβ40 CSF levels among patients with sporadic and hereditary CAA.^15^ We also point out that, in our study, the presence of cSS and a higher imaging burden of CAA were associated with higher total tau protein in CSF, which could reflect a higher severity of neuronal injury and axonal degeneration in this group of patients. Together with these previous results, our findings corroborate in vivo what had been demonstrated in a *post mortem* histopathological study of CAA patients, in which the presence of cerebral hemorrhages was associated with more severe microvascular changes, including vessel wall fragmentation, microaneurysms, and foci of fibrinoid necrosis, but also with more severe vascular amyloid deposition.^16^ Taken together, the results support the pathophysiological timeline of CAA proposed by Koeman and collaborators.^3^

In our cohort, 20% of patients with CAA without hemorrhagic markers had a pathologically elevated albumin ratio according to age. Additionally, CAA patients with hemorrhagic markers had a higher albumin ratio in comparison to patients with no hemorrhagic markers, suggesting a higher degree of blood–brain barrier permeability. Although the mere presence of lobar microbleeds was not associated with albumin ratio, an increasing number of lobar microbleeds was associated with a higher albumin ratio. Likewise, the presence of cSS and a more severe imaging burden of CAA were both associated with higher albumin ratio, thus supporting the hypothesis that blood–brain barrier dysfunction is more severe in more advanced stages of the disease. Although blood–brain barrier leakage appears to be already present in less severe stages of vascular pathology, it increased significantly with the severity of vascular changes in a histopathology study of CAA patients.^17^ Contrary to our results, a previous study showed no association between the presence of cSS and CSF/serum albumin ratio.^18^ However, it was conducted in an unselected population of patients undergoing dementia investigation, only a minority had a diagnosis of CAA, and 17% of patients without cortical superficial siderosis also had lobar microbleeds. Future studies should evaluate the ability of the albumin ratio to predict the occurrence and recurrence of ICH in CAA patients.

Patients with hemorrhagic markers presented less frequently with impairment of episodic memory, and this association was mainly driven by patients with probable CAA according to the Boston version 1.5 criteria. This association must be interpreted with caution and may be explained by the fact that patients with hemorrhagic markers presented significantly less frequently with cognitive impairment. CSF Aβ42/40 ratio was similar in groups with and without hemorrhagic markers. This finding reflects the known pathophysiology of CAA, with deposition not only of Aβ40 in arterial and venous walls but also of Aβ42, which is the predominant form in capillaries.^19^ In contrast to this, a low CSF Aβ42/40 ratio is a well‐known and accepted marker for the diagnosis of Alzheimer's disease because there is a predominant parenchymal deposition of Aβ42. Although a lower CSF Aβ42/40 ratio was associated with the absence of cSS, this association did not persist after the exclusion of patients who fulfilled the diagnostic criteria for Alzheimer's disease. This is probably related to the fact that the group of CAA patients with no or low burden of hemorrhagic lesions was enriched with patients with cognitive presentations and, probably, Alzheimer's disease pathology, leading to a lower CSF Aβ42/40 ratio in this subgroup of patients. On the other hand, higher phosphorylated tau protein in CSF was associated with cSS and more severe imaging burden of CAA after exclusion of patients with Alzheimer's disease, which suggests a complex interplay between amyloid deposition, vascular damage, and neurodegeneration. Similar findings were reported in a previous study of patients with probable CAA, in which both cSS and higher CAA‐SVD score were independently associated with tau pathology in the inferior temporal region in ^18^F‐T807 (tau) PET scans.^20^ Likewise, increasing severity of CAA pathology was associated with increasing tau burden in autopsy studies.^21^, ^22^ We hypothesize that the association we found was only apparent after exclusion of patients with the diagnosis of Alzheimer´s disease, because all of these patients, which in our cohort have a low imaging burden of CAA, had high CSF levels of phosphorylated tau, according to the diagnostic criteria we used.^7^

Surprisingly, we found that patients with CAA with hemorrhagic markers had lower platelet counts than CAA patients without hemorrhagic markers, and this was mainly because of lower platelet counts in patients with possible CAA. Because this association was not previously described, the difference in platelet count is not clinically significant, and patients with probable CAA and CAA patients without hemorrhagic markers had similar platelet counts, the significance of this finding remains unclear.

The main limitations of this study include its retrospective nature, possible underdetection of hospitalized CAA patients related to the use of ICD‐10‐CM codes, and selection bias for performing CSF analysis in patients with suspected CAA, which was decided by the treating physicians and not systematically. The main strengths of this study are the inclusion of a large cohort of CAA patients selected not only from the memory clinic but also from an in‐hospital setting (which enriched our study population with patients with symptomatic ICH and acute convexity subarachnoid hemorrhage) and the availability of both CSF neurodegeneration markers and a marker for blood–brain barrier function. The use of the Boston diagnostic criteria version 2.0 can be discussed as both a strength and a limitation of the study. This is the largest study to date analyzing CSF parameters in CAA patients diagnosed using the Boston diagnostic criteria version 2.0, which allow for the diagnosis of CAA without hemorrhagic markers. However, it is known that the accuracy of the Boston diagnostic criteria version 2.0 is lower for patients without symptomatic ICH,^23^, ^24^ thus possibly inducing bias toward less abnormal CSF parameters in the group without hemorrhagic markers. We expand the findings of two recent studies, which included CAA patients diagnosed using the Boston criteria version 2.0, which found an association of ICH with lower CSF Aβ42 in 31 patients^25^ and with lower CSF Aβ40 in 54 patients.^26^ We emphasize that, in our cohort, there is an overrepresentation of CAA patients assessed in the context of a memory clinic, which increases the validity of our results in this setting. However, most of the previously published studies analyzing CSF markers in patients with CAA focused on hemorrhagic forms, because they were based on the application of the modified Boston criteria version 1.5.

In conclusion, our study provides evidence for different CSF profiles of neurodegeneration markers and blood–brain barrier function parameters in CAA patients with and without hemorrhagic markers and supports the hypothesis of a pathophysiological timeline in CAA. Longitudinal data to understand clinical trajectories of CAA patients without hemorrhagic markers and predictors of progression are needed.

## CONFLICT OF INTEREST STATEMENT

O. Nikoubashman received honoraria for lectures from Werfen. J.B. Schulz received honoraria for lectures and presentations from Eisai, Biogen, and Lilly (payments were made to the RWTH Aachen University); received travel grants from Lilly and Esai; served in the Data Safety Monitoring Board or Advisory Board of Eisai and Lilly. K. Reetz received research grants from the Alzheimer Forschung Initiative e.V. (AFI 13812, NL‐18002CB), the German Federal Ministry of Education and Research (BMBF KP22‐106E), the German Research Foundation (IRTG 2150), Friedreich's Ataxia Research Alliance (FARA) and from the Interdisciplinary Center for Clinical Research within the faculty of Medicine at the RWTH Aachen University; received consulting fees and honoraria for lectures and presentations from Biogen Eisai, Lilly and Roche; is the president of the German Brain Foundation (unpaid position). A.S. Costa received research grants from the Faculty of Medicine RWTH Aachen and from the Alzheimer Forchungsinitative; received honoraria for lectures from Eisai and Lilly. J. Pinho has nothing to disclose. A. Reich has nothing to disclose. Author disclosures are available in the Supporting Information.

## CONSENT STATEMENT

The local ethics committee waived the need for patient‐informed consent because of the retrospective nature of the study.

## Supporting information



Supporting Information

Supporting Information
