# Supplementary material for: Profiles of blood–brain barrier and neurodegeneration markers in cerebrospinal fluid of patients with cerebral amyloid angiopathy
Source: Alzheimers Dement (Amst). 2025 Nov 18;17(4):e70221. doi: 10.1002/dad2.70221 (PMC12626739; doi:10.1002/dad2.70221)
Supplement: Supplementary file 1 — Supporting Information [file DAD2-17-e70221-s002.docx]

Supplementary Table 1. Spearman correlations between age and each of the analysed CFS parameters

|  | **Age (years)** | | |
| --- | --- | --- | --- |
|  | rho | 95% confidence interval | p |
| **Aβ42 (pg/mL)** | -0.20 | -0.30 to -0.10 | <0.001 |
| **Aβ40 (pg/mL)** | 0.03 | -0.08 to 0.14 | 0.545 |
| **Aβ42/40 ratio** | -0.26 | -0.36 to -0.15 | <0.001 |
| **Total tau protein (pg/mL)** | 0.20 | 0.10 to 0.30 | <0.001 |
| **Phosphorilated tau protein (pg/mL)** | 0.18 | 0.07 to 0.28 | <0.001 |
| **Albumin ratio** | 0.01 | -0.11 to 0.12 | 0.925 |

Supplementary Table 2. Multivariable logistic regression analyses with presence of hemorrhagic markers, presence of cerebral lobar microbleeds, presence of cortical superficial siderosis and CAA-SVD score ≥3 as the depend variables, after exclusion of patients with Alzheimer´s disease (n=90).

|  | **Hemorrhage+** | | **Lobar microbleeds** | | **Cortical superficial siderosis** | | **CAA-SVD score ≥3** | |
| --- | --- | --- | --- | --- | --- | --- | --- | --- |
|  | **aOR (95%CI)** | **p** | **aOR (95%CI)** | **p** | **aOR (95%CI)** | **p** | **aOR (95%CI)** | **p** |
| Aβ42 (per 100pg/mL decrease)^*^ | 1.14 (1.04-1.25) | 0.005 | 1.09 (1.00-1.20) | 0.052 | 1.36 (1.15-1.61) | <0.001 | 1.44 (1.23-1.70) | <0.001 |
| Aβ40 (per 1000pg/mL decrease)^*^ | 1.09 (1.02-1.17) | 0.010 | 1.04 (0.98-1.11) | 0.209 | 1.31 (1.16-1.49) | <0.001 | 1.20 (1.09-1.33) | <0.001 |
| Aβ42/40 ratio (per 0.1 point decrease)^*^ | 1.01 (0.94-1.08) | 0.826 | 1.05 (0.97-1.14) | 0.250 | **0.94 (0.87-1.01)** | **0.097** | 1.00 (0.92-1.09) | 0.989 |
| Total tau protein (per 100pg/mL increase)^*^ | **1.13 (1.03-1.25)** | **0.014** | 1.07 (0.98-1.18) | 0.145 | 1.18 (1.06-1.32) | 0.004 | 1.25 (1.11-1.41) | <0.001 |
| Phosphorylated tau (per 10pg/mL increase)^*^ | 1.54 (0.68-3.48) | 0.304 | 1.52 (0.67-3.47) | 0.319 | **2.95 (1.06-8.20)** | **0.038** | **3.05 (1.18-7.85)** | **0.021** |
| Albumin ratio (per 1 point increase)^†^ | 1.13 (1.02-1.25) | 0.016 | 1.08 (0.98-1.19) | 0.111 | 1.15 (1.02-1.30) | 0.020 | 1.16 (1.04-1.30) | 0.011 |

* adjusted for age at lumbar puncture

^†^ adjusted for age at lumbar puncture and serum albumin

aOR: adjusted odds ratio. 95%CI: 95% confidence interval

Results which differ from the main analysis (with patients with Alzheimer´s disease) are marked in bold.

Supplementary Table 3. Multivariable logistic regression analyses with presence of hemorrhagic markers, presence of cerebral lobar microbleeds, presence of cortical superficial siderosis and CAA-SVD score ≥3 as the depend variables, with additional adjustment for presence of antithrombotic therapy at diagnosis.

|  | **Hemorrhage+** | | **Lobar microbleeds** | | **Cortical superficial siderosis** | | **CAA-SVD score ≥3** | |
| --- | --- | --- | --- | --- | --- | --- | --- | --- |
|  | **aOR (95%CI)** | **p** | **aOR (95%CI)** | **p** | **aOR (95%CI)** | **p** | **aOR (95%CI)** | **p** |
| Aβ42 (per 100pg/mL decrease)^*^ | 1.13 (1.04-1.23) | 0.005 | 1.08 (0.99-1.18) | 0.075 | 1.35 (1.14-1.59) | <0.001 | 1.40 (1.19-1.64) | <0.001 |
| Aβ40 (per 1000pg/mL decrease)^*^ | 1.09 (1.04-1.16) | 0.001 | 1.05 (0.99-1.10) | 0.094 | 1.33 (1.20-1.50) | <0.001 | 1.25 (1.14-1.37) | <0.001 |
| Aβ42/40 ratio (per 0.1 point decrease)^*^ | 0.99 (0.93-1.05) | 0.688 | 1.02 (0.95-1.09) | 0.584 | 0.92 (0.86-0.99) | 0.018 | 0.96 (0.89-1.03) | 0.219 |
| Total tau protein (per 100pg/mL increase)^*^ | 1.05 (0.97-1.12) | 0.226 | 0.99 (0.92-1.07) | 0.773 | 1.12 (1.03-1.22) | 0.011 | 1.12 (1.03-1.22) | 0.009 |
| Phosphorylated tau (per 10pg/mL increase)^*^ | 1.06 (0.58-1.93) | 0.846 | 0.95 (0.51-1.75) | 0.860 | 1.57 (0.70-3.52) | 0.269 | 1.50 (0.71-3.17) | 0.286 |
| Albumin ratio (per 1 point increase)^†^ | 1.12 (1.03-1.22) | 0.009 | 1.07 (0.99-1.17) | 0.089 | 1.14 (1.03-1.27) | 0.011 | 1.17 (1.06-1.30) | 0.003 |

* adjusted for age at lumbar puncture and antithrombotic medication (antiplatelet and/or anticoagulation) at the time of diagnosis

^†^ adjusted for age at lumbar puncture, antithrombotic medication (antiplatelet and/or anticoagulation) at the time of diagnosis and serum albumin

aOR: adjusted odds ratio. 95%CI: 95% confidence interval
